# Improved Subthreshold Characteristics by Back-Gate Coupling on Ferroelectric ETSOI FETs

**DOI:** 10.1186/s11671-022-03767-4

**Published:** 2022-12-15

**Authors:** Zhaohao Zhang, Yudong Li, Jing Xu, Bo Tang, Jinjuan Xiang, Junjie Li, Qingzhu Zhang, Zhenhua Wu, Huaxiang Yin, Jun Luo, Wenwu Wang

**Affiliations:** 1 Key Laboratory of Microelectronics Devices and Integrated Technology, Institute of Microelectronics, CAS, Beijing, China; 2 Changcheng Institute of Metrology & Measurement, Beijing, China; 3 School of Integrated Circuits of University of Chinese Academy of Sciences, Beijing, 100049 China

**Keywords:** Hf_0.5_Zr_0.5_O_2_, ETSOI, Back gate, Subthreshold swing, Domain switching

## Abstract

In this work, extremely thin silicon-on-insulator field effective transistors (ETSOI FETs) are fabricated with an ultra-thin 3 nm ferroelectric (FE) hafnium zirconium oxides (Hf_0.5_Z_r0.5_O_2_) layer. Furthermore, the subthreshold characteristics of the devices with double gate modulation are investigated extensively. Contributing to the advantages of the back-gate voltage coupling effects, the minimum subthreshold swing (SS) value of a 40 nm ETSOI device could be adjusted from the initial 80.8–50 mV/dec, which shows ultra-steep SS characteristics. To illustrate this electrical character, a simple analytical model based on the transient Miller model is demonstrated. This work shows the feasibility of FE ETSOI FET for ultra-low-power applications with dynamic threshold adjustment.

**Background**

To fabricate an ultra-low-power CMOS integrated circuits, many researches have investigated field effect transistors (FETs) with new structures [1–3] or revolutionary principles [4, 5]. Extremely thin silicon-on-insulator (ETSOI) FETs, which have improved the gate control ability and reduced the leakage by the fully depleted channel and bottom isolation, respectively, are proposed to realize ultra-low-power-consumption circuits [6–15]. However, the devices cannot break the limitations of “Boltzmann tyranny” only by structural innovation. Ferroelectric ETSOI FETs (FE ETSOI FET), which integrate a ferroelectric (FE) film into the gate stacks, could realize the amplification of the surface voltage on the channel and achieve super-steep SSs (< 60 mV/dec) [16–18]. In the past several years, the improved subthreshold characteristics of the FE ETSOI FETs were reported [19, 20]. Although the subthreshold characteristics could be improved, there are few works revealing the back-gate coupling effect on the performances of the FE devices.

In this work, ETSOI FETs are fabricated with an ultra-thin 3-nm-thick FE hafnium zirconium oxides (Hf_0.5_Zr_0.5_O_2_) film. Based on the advantages of double gate structure of the devices, a method for improving the subthreshold characteristics of FE ETSOI FETs by back-gate voltage coupling is demonstrated. The values of the subthreshold swing (SS) could be adjusted from 80.8 to 50 mV/dec by the back-gate voltage modulating for a 40 nm physical gate length (L_G_) ETSOI device, which shows obvious ultra-steep SS characteristics. To illustrate this electrical character, a simple analytical model based on the transient Miller model is used in this work.

## Method

**Fig. 1:**
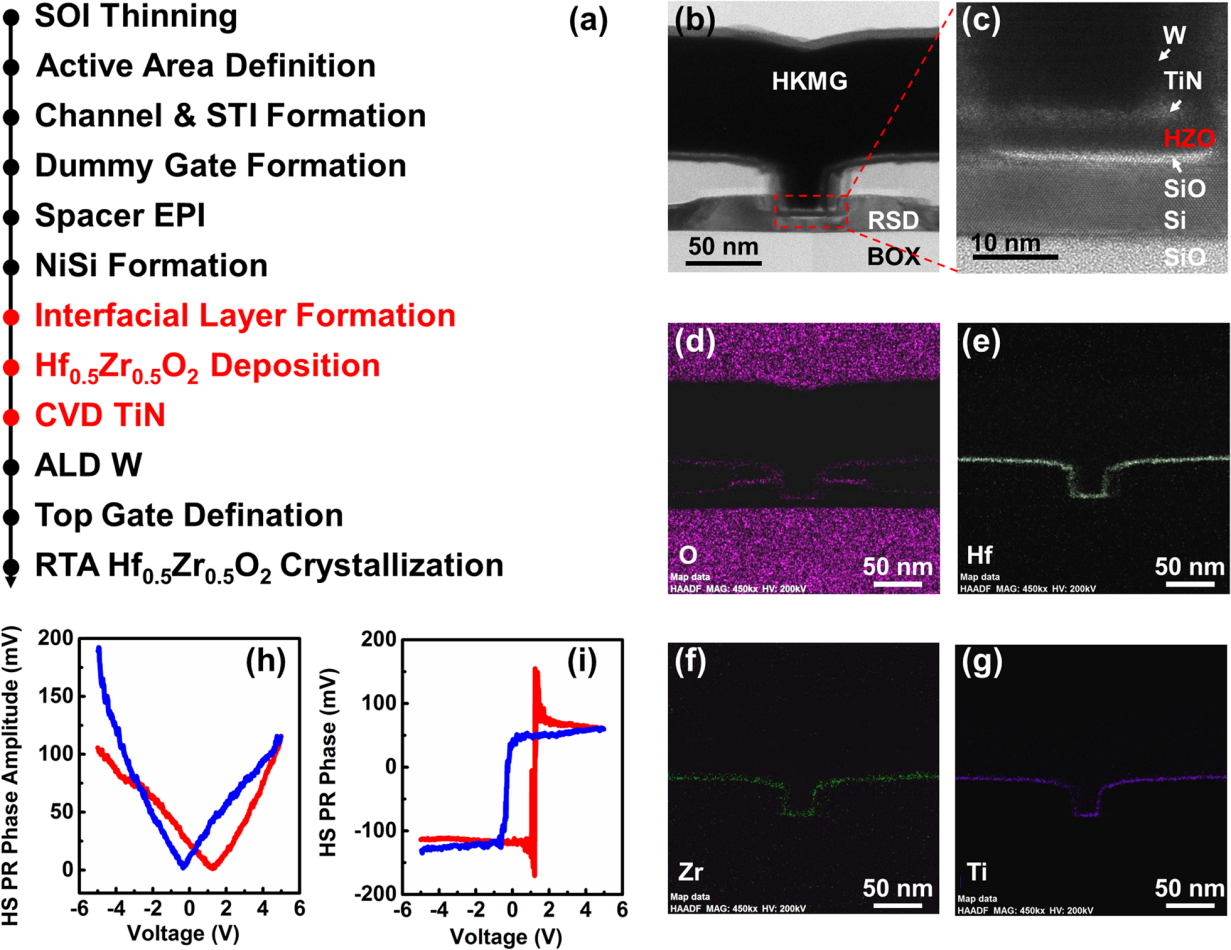
(**a**) Process flow of Hf_0.5_Zr_0.5_O_2_ ETSOI *n*-type MOSFETs fabrication, (**b**) TEM cross-sectional image for Hf_0.5_Zr_0.5_O_2_ ETSOI MOSFETs with raised source/drain structure and ultra-thin channel, (**c**) enlarged image of the gate stacks in (**b**). (**d**) O, (**e**) Hf, (**f**) Zr and (**g**) Ti elements distribution of the ETSOI MOSFETs. (**h**) PFM phase and (**i**) amplitude hysteresis loops taken on the TiN top electrode with 3 nm Hf_0.5_Zr0_.5_O_2_

Devices were fabricated on SOI wafers with a buried oxide (BOX) thickness of 145 nm. A process fabrication flow of the ETSOI MOSFET is depicted in Fig. 1a. The ultra-thin top Si layers of the SOI substrates are thinned to 7 nm by thermal oxidation followed by diluted hydrofluoric acid. Dummy poly gates were formed followed by ultra-thin spacers (∼8 nm). Faced raised source and drain (RSD) was epi-grown with in situ doped boron ions. In order to form high-quality raised SiGe SD, the thickness of silicon loss in SD area needs to be carefully controlled. In the flowing steps, an additional implantation of As and a rapid thermal anneal (RTA) process was performed to drive in the doped ions to form the extensions. After self-allied silicide formation, dummy poly gates were removed. In the flowing steps, after the ~ 1 nm SiO2 interfacial layer (IL) formation by chemical O_3_ oxidation, a sequential deposition of multilayer Hf_0.5_Zr_0.5_O_2_ and TiN films was performed by an atomic layer deposition (ALD) and chemical vapor deposition (CVD) process, respectively, where the FE Hf_0.5_Zr_0.5_O_2_ material replaced the conventional HfO_2_ film. The Hf_0.5_Zr_0.5_O_2_ film (3 nm) was deposited by ALD at 300 °C using Hf (TEMAH) and Zr (TEMAZ)-based organic precursors. A RTA process of 550 °C/30 s at nitrogen atmosphere was carried out after the deposition of Hf_0.5_Zr_0.5_O_2_, which was also helpful to improve the quality of the Hf_0.5_Zr_0.5_O_2_ film. The SD metal contact by the W-plug and the alloy processes by forming gas annealing (FGA) at 450 °C/30 min were carried out in the subsequent steps. The cross-sectional profiles of FE ETSOI FET were observed using a cross-sectional transmission electron microscope (TEM). The electrical characterization was performed using Keithley 4200 and Agilent 4156C semiconductor parameter analyzers.

## Result and Discussion

Figure 1b shows the cross-sectional TEM image of a 40-nm L_G_ device. High-κ metal gates (HKMGs) multilayers, W/3-nm TiN/3-nm HZO/1-nm SiO (IL), are shown in Fig. 1c. In this figure, multilayer HKMGs distributed on the channel are highly conformal and uniform. The thicknesses of the IL and FE layers, labeled in Fig. 1c, are about 1 nm and 3 nm, respectively, and the ultra-thin Si channel layer is 7 nm in thickness, which contributes to better control of the short channel effects (SCEs) than that of bulk MOSFET. Figure 1d–g show the elements distribution of the FE device. It can be seen that Hf and Zr atoms are basically concentrated in the medium layer, and there is no diffusion for multilayer materials. The above results show that the process controls appropriate during manufacturing process of the device, and the Hf_0.5_Zr_0.5_O_2_ has good process compatibility with the conventional CMOS fabrication.

Furthermore, to confirm the ferroelectricity of the HZO film, capacitors with TiN /3-nm HZO/ ~ 1-nm SiO_2_/Si were fabricated, which own similarly conditions with the devices, and analyzed by piezoresponse force microscopy (PFM) test using Asylum MFP-3D. Figure 1h and i show corresponding results for the characterization with a typical frequency range ~ 350–400 kHz. The presence of square 180° hysteresis in PFM phase indicates the upward and downward polarization states, and butterfly-shaped loops in PFM amplitude implies robust remanent polarization for the FE film.

**Fig. 2:**
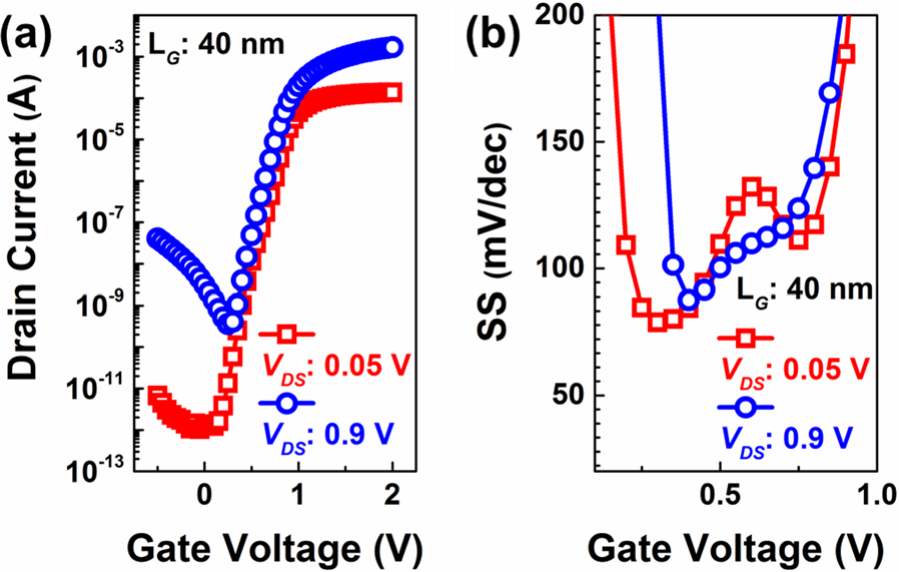
(**a**) Transfer curves of the device with *L*_*G*_ = 40 nm, *W* = 8 μm. (**b**) Extracted SSs as a function of the *V*_*GS*_ of the device

Figure 2a shows the transfer curves (*I*_*DS*_*–V*_*GS*_) of a 40-nm L_G_ FE ETSOI FETs at *V*_*DS*_s of 50 and 900 mV, respectively. The calculated value of the drain-induced barrier lowering (DIBL) is ~ 130 mV/V indicating serious SCEs. In addition, Fig. 2b summarizes the corresponding variations of SSs as a function of *V*_*GS*_ for the devices. The results show that the minimum value of the SS is higher than 82 mV/dec, which is much higher the limit value of “Boltzmann tyranny.”

**Fig. 3:**
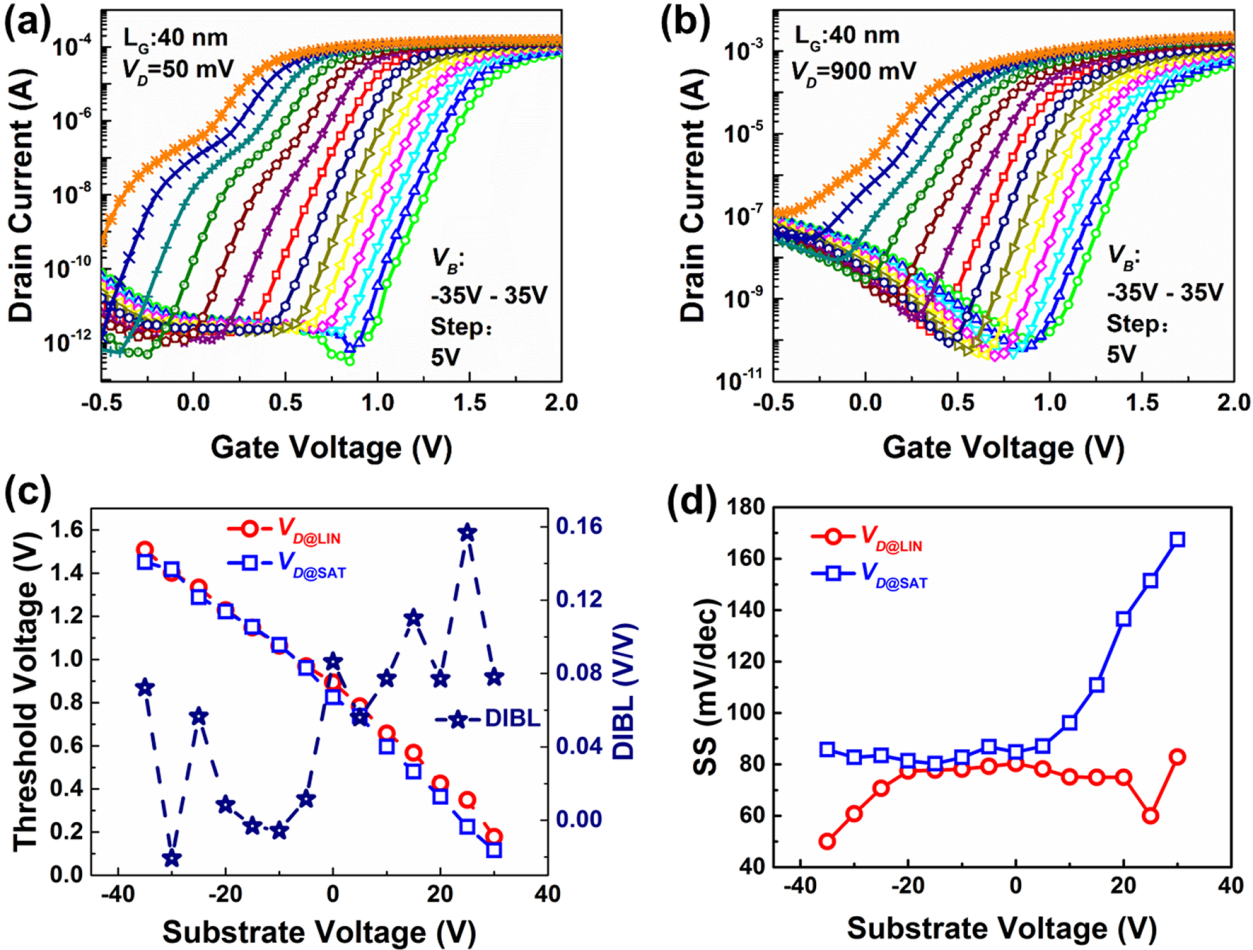
Impacts of back bias voltages on transfer curves of FE ETSOI FET, with (**a**) *V*_*DS*_ = 0.05V, and (**b**) *V*_*DS*_ = 0.9 V, with *L*_*G*_ = 40 nm. (**c**) Extracted *V*_*T*_s and DIBLs under various *V*_*SUB*_s of the device in Fig. 3a. (**d**) Extracted SSs under various *V*_*SUB*_s of the device in Fig. 3a and (b).

In order to improve the SCEs, based on the advantages of double gate structure of the devices, a method for enhancing the subthreshold characteristics by back-gate voltage coupling effects is demonstrated and analyzed, subsequently. The *I*_*DS*_*–V*_*GS*_ of a 40-nm *L*_G_ device at *V*_*DS*_ = 50 mV (*V*_*DS@LIN*_) and 900 mV (*V*_*DS@SAT*_) with various bias gate voltages (*V*_*SUB*_) from −35 to 35 V are shown in Fig. 3a and b, respectively. When the value of *V*_*SUB*_ bias is positive, there is an obvious shoulder near *I*_*DS*_ = 10^–8^ A. But when the value of *V*_*SUB*_ bias is changed to negative, the shoulder disappears. This indicates that the back channel of the device has a parasitic device which is also controlled by *V*_GS_. The positive bias will enhance the parasitic effect, while the negative bias can turn off the parasitic effect.

Figure 3c summarizes the corresponding variations of threshold voltage (*V*_*T*_s) and factors of DIBLs as a function of *V*_*SUB*_s for the device shown in Fig. 3a. All of the corresponding *V*_*T*_ values are extracted at a fixed normalized *I*_DS_ of 10 nA/μm. It can be seen that both *V*_*T@LIN*_ and *V*_*T@SAT*_ increase linearly with *V*_*SUB*_ changing from positive to negative. With different bias of the *V*_*SUB*_, the *V*_*T*_ can be tuned within the range of almost 1.5 V. It demonstrates *V*_*SUB*_ bias can be effectively served as a method to modulate ETSOI device characteristics due to the controlling of carrier confinement. Furthermore, it is worth noting that with the decrease of *V*_*SUB*_, the difference between *V*_*T@LIN*_ and *V*_*T@SAT*_ tends to decrease gradually, which means smaller values of the DIBLs and the improved SCEs.

With V_SUB_ value decreasing continuously, the value of *V*_*T@LIN*_—*V*_*T @SAT*_ shows a decrease tendency and changed from positive under + *V*_*SUB*_ to negative under some -*V*_*SUB*_ value, which indicates the phenomenon of the negative DIBL (N-DIBL). With various *V*_*SUB*_ bias, the behaviors of SCEs are different. For positive *V*_*SUB*_ bias, currieries were pulled away from top channel, and thus, DIBL gets worse. Besides, for negative *V*_*SUB*_ bias, top gate controllability of channel carriers was enhanced, thereby achieving better DIBL performance. Furthermore, the random variation of DIBLs, especially for those negative DIBLs, may be caused by the transit negative capacitance phenomenon induced by the HZO film in the gate stacks [21]. In addition, Fig. 3d summarizes the corresponding variations of SSs as a function of *V*_*DS*_ for the devices. Corresponding to the above results, with the decrease of *V*_*SUB*_, the values of SSs show a decreasing trend and the minimum SS value of the device reaches 50 mV/dec at *V*_*DS*_ = 50 mV and *V*_*SUB*_ =  − 35 V, which is far below the limit of 60 mV/dec for the conventional FETs and exhibiting the great advantage for ultra-low-power application. Due to the shoulder existing under high positive *V*_*SUB*_ with 50 mV *V*_*D*_, the accurate minimum SS values should be higher than that shown in Fig. 3d, but it does not affect the conclusion that subthreshold characteristics improving under negative *V*_*SUB*_.

**Fig. 4:**
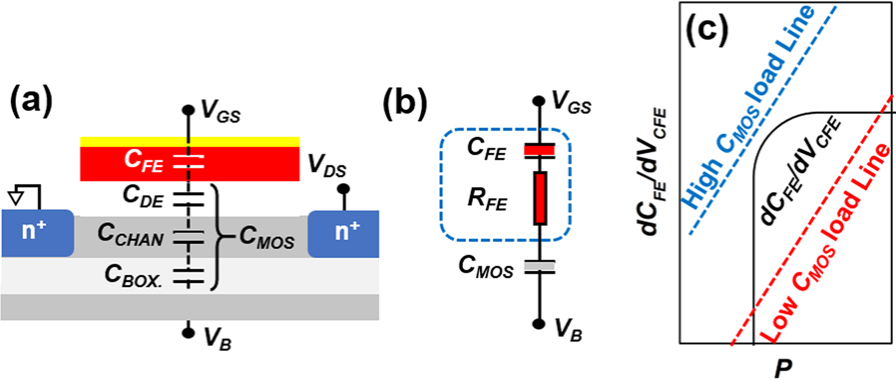
(**a**) Capacitance in FE ETSOI MOSFET. (**b**) Equivalent circuit model of DE–FE network according to Miller model. (**c**) Graphical representation of the equation with respect to P for different values of *C*_*MOS*_.

To illustrate the mechanism of the substrate voltage enhancing SCEs of FE ETSOI FET, a simple analytical model based on ferroelectric domain switching is used shown in Fig. 4. Figure 4a shows capacitance coupling in fabricated FE ETSOI FET, where *C*_*FE*_, *C*_*DE*_, *C*_*CHAN*_ and *C*_*BOX*_ are capacitance of FE film, silicon oxide dielectric layer, silicon channel and box silicon oxide, respectively. *C*_*MOS*_ can be written as (*C*_*MOS*_^−1^ = *C*_*DE*_^−1^ + *C*_*CHAN*_^−1^ + *C*_*BOX*_^−1^). According to the transient Miller model [22, 23], the equivalent circuit model of this DE–FE network is shown in Fig. 4b. Considering current flowing through the FE–DE stack, the equation for *V*_*RFE*_ as *V*_*RFE*_ = *R*_*FE*_ × *I* = (τ/*C*_*FE*_) × *I* = (τ/*C*_*FE*_) × *C*_*FE*_ (*dV*_*CFE*_*/dt*) = τ × (*dV*_*CFE*_*/dt*) and *V*_*RFE*_ = [τ × *C*_*DE*_/(*C*_*DE*_ + *C*_*FE*_)] × [*dV*_*GS*_*/dt- dV*_*FE*_*/dt* + *dV*_*CFE*_*/dt*] can be written. Here, *V*_*FE*_ and *V*_*RFE*_ are voltage drops across *C*_*FE*_ and *R*_*FE*_, and τ is the lag between *V*_*FE*_ and the polarization (*P*). After differentiating these two equations with respect to time (t), an equation for *dV*_*FE*_*/dt* can be derived. For ultra-steep SS characteristics, *dV*_*FE*_*/dt* should change from a positive to a negative value in case of positive ramp (*dV*_*GS*_*/dt* and *dP/dt* are positive) or negative to positive for negative ramp. Therefore, the condition for ultra-steep SS characteristics in the DE–FE network can be written as the following equation:

$$\left|\frac{{dC}_{FE}}{d{V}_{CFE}}\right|>{\left[1-\frac{d\tau }{dt}\right]\left[\frac{\tau }{CMOS+CFE}\left|\frac{{dV}_{GS}}{dt}\right|*\left|\frac{{dC}_{FE}}{dt}\right|\left(\frac{{dV}_{CFE}}{d{V}_{GS}}\right)\right]}^{-1}$$

This equation suggests that the increase in *C*_*FE*_ with *V*_*CFE*_ is the prime factor for the ultra-steep SS characteristics, which must be sufficiently large. The discussion in this work assumes that τ, *dC*_*FE*_*/dt* and *dV*_*CFE*_*/dV*_*GS*_ are not affected by *V*_*SUB*_ changing, so we can conclude from the equation that ultra-steep SS will be easier to meet with decrease in *C*_*MOS*_.

For the fabricated FE ETSOI MOSFET, as *V*_*SUB*_ increases, the energy band of the channel gradually bends, that is to say, more electrons could fill the channel. Due to the charge shielding effects of the filled electrons, the *C*_*MOS*_ value gradually increases. As Fig. 4c shown, with a low *C*_*MOS*_ value, the inequality is easier to establish, and thus, the ultra-steep SSs are more easily to be obtained [22].

## Conclusion

In this work, an innovative way to enhance the subthreshold characteristics effects by coupling back-gate voltage is proposed and realized initially with FE ETSOI MOSFETs. The minimum SS value of a 40 nm ETSOI device could be adjusted from the initial 80.8 to 50 mV/dec, which shows ultra-steep SS characteristics. A simple analytical model based on the transient Miller model is used in this work to illustrate the mechanism. This work demonstrates the feasibility of FE ETSOI MOSFET in ultra-low-power application with dynamic threshold adjustment.

## Data Availability

The datasets supporting the conclusions of this article are included in the article.

## Abbreviations

ETSOI FETs: Extremely thin silicon-on-insulator field effective transistors
FE: Ferroelectric
Hf_0.5_Zr_0.5_O_2_: Hafnium zirconium oxides
SS: Subthreshold swing
IL: Interfacial layer
ALD: Atomic layer deposition
CVD: Chemical vapor deposition
TEM: Transmission electron microscope
L_G_: Gate length
HKMGs: High-κ metal gates
SCEs: Short channel effects
PFM: Piezoresponse force microscopy
*I*_DS_–*V*_GS_: Transfer curves
DIBL: Drain-induced barrier lowering
N-DIBL: Negative DIBL
